# Electrophysiological Study of the Masticatory Muscle Activity in Patients With Temporomandibular Disorders With and Without Tinnitus

**DOI:** 10.1002/cre2.70172

**Published:** 2025-07-13

**Authors:** Henri Albert Didier, Alberto Maria Cappellari, Federica Di Berardino, Margherita Donelli, Xi Xi Fan, Laura Magnoni, Giorgio Lilli, Stefania Barozzi, Diego Zanetti, Flavio Arnone, Alexandre Henri Didier, Gennaro Bussone, Alberto Caprioglio, Aldo Bruno Giannì

**Affiliations:** ^1^ Istituto Stomatologico Italiano Milan Italy; ^2^ Neuromuscular Gnathology Tutor – University of Milan Milan Italy; ^3^ Department of Neuroscience Fondazione IRCCS Ca' Granda Ospedale Maggiore Policlinico Milan Italy; ^4^ Department of Clinical Sciences and Community Health University of Milan Milan Italy; ^5^ Audiology Unit, Department of Specialist Surgical Sciences Fondazione IRCCS Ca' Granda Ospedale Maggiore Policlinico Milan Italy; ^6^ Resident in the Full‐time Post‐graduate Program in Orthodontics, Department of Biomedical, Surgical and Dental Sciences University of Milan Italy; ^7^ Lean Six Sigma Politecnico di Milano Milan Italy; ^8^ Operations and Supply Chain Management University of Liverpool UK; ^9^ Azienda Ospedaliera Santi Paolo e Carlo Milan Italy; ^10^ Casa di Cura del Policlinico Igea Headache Center Milan Italy; ^11^ Department of Biomedical, Surgical and Dental Sciences University of Milan Milan Italy; ^12^ Maxillo‐Facial Surgery and Dental Unit Fondazione IRCCS Ca' Granda Ospedale Maggiore Policlinico Milan Italy

**Keywords:** audiology, mandibular rest position, surface electromyography, TENS, tinnitus, TMJ disorders

## Abstract

**Objectives:**

The relationship between tinnitus and temporomandibular disorders (TMD) over the years has continued to be widely debated in literature and not yet fully understood. Several causes have been associated with tinnitus including altered mandibular posture and disorders at the muscle, joint and periodontal receptors. This study aimed to investigate masseter and temporalis muscle activity in patients with TMD with and without somatosensory tinnitus (ST).

**Material and Methods:**

A total of 105 patients (mean age 50.49 ± 11.7 years) with TMD classified according to Axis I DC/TMD diagnostic criteria were enrolled and then divided into two groups: 53 subjects with ST and 52 without ST. Electromyographic and kinesiographic instrumental examinations were used for data collection. Statistical analyses, including Levene's test and independent *t*‐test, were performed to evaluate and compare muscle activity abnormalities between the two groups.

**Results:**

The study showed a higher incidence of abnormal masticatory muscle activity in habitual resting position in TMD without ST patients compared to those with TMD and ST. A statistically significant difference in muscle activity between the two groups was observed.

**Conclusions:**

The results suggest a potential link between masticatory muscle activity and tinnitus in TMD patients. Neuroplasticity may play a role in modulating the psychoacoustic characteristics of tinnitus.

## 1 Background

Tinnitus, defined as the perception of sound in the absence of an external auditory source, is a common and complex condition with a prevalence ranging from 5% to 15% in the adult population (Fabrizia d’ et al. 2025; Jarach et al. 2022). Its etiology is multifactorial. Tinnitus can be influenced by head, neck, and jaw movements, and somatosensory inputs can modulate neural activity in the cochlear nucleus (Wu et al. 2016).

Somatosensory tinnitus (ST) is a specific subtype of tinnitus, where somatosensory input from the upper cervical spine or temporomandibular area influences the tinnitus percept through existing brainstem connections (Levine 1999; Levine and Oron 2015). Several studies indicate that cervical and temporomandibular somatosensory signals reach the brain through afferent fibers, whose cell bodies are located in the dorsal root ganglia or the trigeminal ganglion. Some of these fibers also project to the central auditory system, allowing somatosensory input to modulate the auditory system by influencing spontaneous rates or synchrony of firing among neurons in the cochlear nucleus, inferior colliculus, or auditory cortex. As a result, the somatosensory system can impact tinnitus perception by modifying its pitch or loudness (Shore 2011; Shore et al. 2007; Ralli et al. 2016). Growing evidence indicates a significant association between temporomandibular disorders (TMD) and ST (Vielsmeier et al. 2011; De La Torre Canales et al. 2024; Didier et al. 2023; Prado et al. 2024). TMD are a group of musculoskeletal and neuromuscular conditions affecting the temporomandibular joint (TMJ), masticatory muscles, and associated structures (Wieckiewicz et al. 2022). TMD affects approximately 34% of the global population. The etiology of TMD is multifactorial, including stories of trauma, anatomical variations, biological, psychological, and social factors (Wieckiewicz et al. 2022; Orzeszek et al. 2023; Cigdem Karacay and Sahbaz 2023). The Diagnostic Criteria for TMD (DC/TMD) (Schiffman et al. 2014) provides a standardized framework for assessing both the biological and psychosocial components of these disorders. A common factor associated with TMD is bruxism, which involves involuntary teeth grinding or jaw clenching, either during sleep or while awake. Bruxism is believed to contribute to TMD by increasing muscle tension and joint stress. Recent data show that TMD and bruxism often co‐occur, with estimates suggesting a global associated rate of around 17%, while the global prevalence of bruxism was estimated at 22% (Zieliński et al. 2024, 2025).

Clinically, the major signs and symptoms related to TMD are pain in the TMJ and masticatory muscles, impaired jaw movements, TMJ sounds, and otologic symptoms such as hearing loss, vertigo, dizziness, ear pain, and tinnitus (Bair et al. 2016; Slade et al. 2016).

It has been seen that individuals with TMD have faced the chance of experiencing tinnitus up to almost 5 times more than those without TMD; moreover, the prevalence of TMD among patients with ST is significantly higher than the prevalence of ST among patients with TMD (Mottaghi et al. 2019).

Many causes have been suggested to understand this correlation, including disorders of muscular, articular, and periodontal receptors (Levine 1999). Altered mandibular posture, resulting in abnormal activity of all these receptors, appears to be a trigger, predisposing, or perpetuating factor of tinnitus (Ganz Sanchez et al. 2002).

The influence of muscular tension associated to parafunction or occlusal disorders on the rest mandible position could explain the usefulness of transcutaneous electrical nerve stimulation (TENS) in identifying the physiologic mandibular posture, as well as the discrepancies of open‐close trajectory in the sagittal and frontal planes in patients with TMD compared to normal subjects (Haider et al. 2018; Michiels et al. 2018; Cooper 2004). Although there are many neurophysiologic studies on masticatory muscles in patients with TMD, their usefulness in patients with ST is less clear (Hugger et al. 2012; Cardoso et al. 2020; Santana‐Mora et al. 2009; Zieliński et al. 2019).

To the best of our knowledge, no previous studies had analyzed ST and mandibular posture through surface electromyographic (sEMG) and kinesiographic analysis.

This paper aims to explore the association between TMD and tinnitus, with a particular focus on the masseter and anterior temporal muscles' activity and the presence of an altered position of the mandible before and after TENS, using sEMG and Kinesiography, in patients affected by TMD compared to those with TMD and ST.

Understanding these interactions might contribute to having an objective rather than subjective (Sanchez and Rocha 2011a; Michiels et al. 2018) point of view and improving clinical management and therapeutic outcomes for affected patients.

## 2 Methods

### 2.1 Study Design

The Strengthening the Reporting of observational studies in Epidemiology Statement (STROBE) checklist for observational studies was used to structure and organize the present study (von Elm et al. 2008). The study included two groups of patients affected by TMD, respectively, with and without tinnitus. All subjects were investigated at the Audiology and Odontostomatology Units of the Fondazione IRCCS Ca' Granda, Ospedale Maggiore Policlinico, Milan, and Istituto Stomatologico Italiano ISI, Milan, Italy, after obtaining signed informed consent. A unique identification code was assigned to each patient to ensure the anonymity of personal data. All medical information contained in the medical records was available only to authorized medical personnel. This study was performed according to the Declaration of Helsinki for human studies. The protocol was approved by the Ethical Committee of the IRCCS Fondazione Ca' Granda, Ospedale Maggiore, Milan (Protocol No.: 653).

### 2.2 Type of Participants and Inclusion Criteria

A total of 105 patients, aged between 23 and 75 years (mean age: 50.49 ± 11.7 years), were enrolled in the study. Participants were divided into two groups: the first group included 53 patients (23 males and 30 females, mean age: 49.51 ± 12.85 years) diagnosed with TMD associated with ST (ST group), while the second group, used as controls, comprised 52 patients (23 males and 29 females, mean age: 51.48 ± 10.44 years) diagnosed with TMD but without ST (w/o‐ST group). Patients included in the ST group were selected from those referred to the audiology clinic with a diagnosis of ST, confirmed according to current guidelines (Haider et al. 2018; Michiels et al. 2018; Attanasio et al. 2015; Sanchez and Rocha 2011b).

However, to ensure the accuracy of the sample, individuals whose tinnitus was associated with hearing loss, Ménière's disease, vertigo, middle ear, or intracranial pathologies were excluded. Patients with a history of whiplash injury or previous cervical spine surgery were also excluded from the study. Furthermore, inclusion in the ST group required a concomitant diagnosis of TMD, which was confirmed through a detailed stomatological examination. Only patients with a confirmed diagnosis of both TMD and ST were included in this group.

Patients in the w/o‐ST group were initially evaluated at the stomatological clinic due to symptoms related to the temporomandibular joint. To ensure the absence of otologic diseases or hearing loss, all patients in this group subsequently underwent assessment at the audiology clinic. Exclusion criteria common to both groups included the presence of neuromuscular and/or degenerative diseases, a history of trauma in the TMJ area, autoimmune diseases affecting the joints, prior treatment for TMD, pregnancy, infections, neoplasms, and prior cervical physical rehabilitation treatments. The history of the pain‐related TMD was collected, asking about the presence/absence of masticatory system pain, headache of any type in the temporal region, pain or headache modification with jaw movement, function, or parafunction.

Clinical assessment was conducted according to the diagnostic criteria for Axis I temporomandibular disorders (DC/TMD) (Schiffman et al. 2014), which classify patients into different diagnostic subgroups and analyze patients' pain‐related TMD. The assessment included myofascial pain (1a), myofascial pain with limited mouth opening (1b), disc displacement with reduction (2a), disc displacement without reduction with limited opening (2b), disc displacement without reduction and without limited opening (2c), arthralgia (3a), osteoarthritis (3b), and osteoarthrosis (3c). Patients with ST fell into diagnostic groups 1a and 2a, whereas those without ST were classified as 1a, 1b, 2a, 2b, 2c, and 3a. All participants completed a standardized questionnaire to collect information regarding psychological aspects and TMJ symptoms, including joint noise, joint pain, and functional limitation (Ohrbach 2016).

### 2.3 Data Acquisition Protocol

Our study was based on Kinesiography and Electromyography. These are two procedures based on a computerized examination designed by Bernard Jankelson(Jankelson et al. 1975), aiming to investigate the mandibular kinematics and muscle activity, as well to record the physiological mandibular trajectory during TENS neurostimulation. The instrumentation used (Myotronics/Normed Inc., Tukwila, Washington) consisted of a K7/EMG electromyograph, a K7/CMS kinesiograph with associated magnet, and a TENS J4 Myomonitor unit with Myotrode SG monopolar electrodes.

The patients underwent surface K7/EMG electromyographic examination to assess the activity of the masticatory muscles in the habitual resting position of the mandible.

To ensure reproducibility of the surface electromyography (sEMG) procedure used in dentistry, especially when assessing masticatory muscle activity, it is essential to adhere to a standardized methodology that addresses every phase of the data acquisition process, as already suggested by Zieliński and Gawda (2024) (Zieliński and Gawda 2024).

The process begins with standardized patient preparation. The skin should be cleansed using 70% isopropyl alcohol to minimize impedance and enhance signal quality, as outlined by Konrad (Konrad 2005). It is also essential guarantee a correct posture of the patient during examination. Subjects should be seated upright on a chair with back support, with hands resting on their legs, eyes closed, and jaw in its most relaxed position. This posture reduces involuntary muscle activation and external artifacts.

Electrode placement must follow established guidelines, such as those proposed by the SENIAM (Hermens et al. 2000) project, which provide recommendations for precise electrode positioning over muscles like the masseter and temporalis (Hermens et al. 2000). Specifically, bipolar Ag/AgCl electrodes should be used, spaced approximately 20 mm apart to optimize signal detection while minimizing crosstalk. A sampling frequency of at least 2000 Hz is recommended to comply with the Nyquist–Shannon theorem and to capture the full range of muscle signal frequencies (Smalt and Brungart 2022; Durkin and Callaghan 2005). The bandwidth should be set between 20 and 500 Hz, to capture both slow tonic and fast phasic muscle activities.

High input impedance (≥ 100 MΩ), a strong common mode rejection ratio (≥ 100 dB), and low baseline noise (< 5 μV RMS) are also essential to ensure signal clarity. Filters must be appropriately applied, by using a high‐pass filter of 20 Hz to reduce low‐frequency noise due to movement, and a low‐pass filter of 500 Hz to remove electrical interference. The importance of appropriate filtering in sEMG analysis has been underscored by recent studies, such as that of Karacan et al. (2023).

During data collection, the muscle activity of the right masseter muscle (RMM), left masseter (LMM), right anterior temporalis (RTA), and left anterior temporalis (LTA) were investigated. Myotronics K7/EMG allow to analyze the following masticatory muscles: TA—the temporalis anterior muscle; MM—the superficial part of the masseter muscle; DA—the anterior belly of the digastric muscle; SCM—the middle part of the sternocleidomastoid muscle. In this study, we decided to assess only TA and MM muscles because, when relaxed with TENS, they can provide a physiological mandibular rest position and physiological mandibular trajectory (Chipaila et al. 2014).

**Figure 1:**
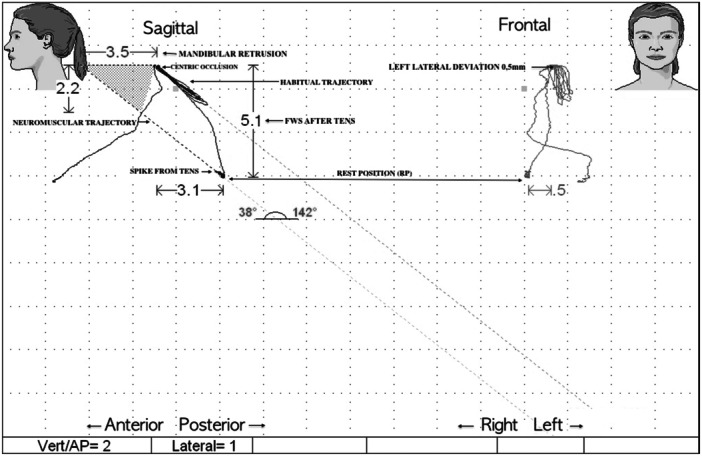
A Scan 5 example, showing the registration of the discrepancy between the habitual closing trajectory and that induced by TENS (neuromuscular trajectory) in the sagittal and frontal planes. In this patient, the trajectory is retruded by 3.5 mm with a Free Way Space of 5.1 mm, along with a left lateral deviation of 0.5 mm.

Finally, the usual resting muscle activity was recorded (SCAN 9). In addition, a K7/CMS Kinesiograph examination was performed to assess and record any discrepancies in the sagittal (posterior displacement) and frontal (lateral displacement) planes (SCAN 5) between the habitual and TENS‐induced closure trajectory (neuromuscular trajectory), as shown in Figure 1.

### 2.4 Statistical Analysis

A priori sample size calculation was performed using G*POWER (version 3.1.9.7) (Faul et al. 2009, 2007) to reject the null hypothesis of equal population means between the ST and w/o ST groups. For a two‐tailed *t*‐test the Cohen's d effect size was calculated (Sullivan and Feinn 2012).

To compare the level of muscular abnormalities between ST group and w/o‐ST group, we rearranged the reported data by attributing to each variable a level of abnormality. As reported by Cooper (2004) and Wang et al. (2025), the EMG values of the masticatory muscles in habitual rest position were considered abnormal if ≥ 2.8 μV for RTA and LTA muscles and ≥ 2.3 μV for the RMM and LMM muscles. In the kinesiographic study, lateral displacements ≥ 0.4 mm were considered abnormal, as well as sagittal displacements ≥ 2 mm (Campillo et al. 2017; Cardoso et al. 2020). A score was given to each individual subject based on the number of muscles that showed abnormal muscle activity in the habitual rest position, ranging from 1 (no abnormal activity) to 5; including 2 (only one muscle with abnormal activity), 3 (two muscles), 4 (three muscles), and 5 (four muscles). As all subjects demonstrated lateral displacement, no scores were attributed in this regard, as it does not affect the evaluation results of these two samples. Based on the abnormalities scores, Levene's test was used to assess whether the variances of the two groups analyzed were homogeneous. Then, an independent *t*‐test with equal variances was performed using Stata 18 software (StataCorp 2023). In the statistical analyses, both *p*‐values and corresponding effect sizes were reported, and the significance level was set to *p* < 0.05. The null hypothesis defines that the means of muscle activity abnormalities between the two groups are equal, while the alternative hypothesis states that these means are different.

## 3 Results

The results of G*POWER suggested that a sample size of 40 subjects per group (*N* = 80) was required Assuming an independent *t*‐test, an effect size (Cohen's d) of 0.824, a type I error probability (α) of 0.05, and a statistical power (1 – β) of 0.95. This high power reflects a strong likelihood of identifying real differences between the groups, provided that the assumed effect size accurately reflects the population.

The sociodemographic characteristics of the sample are reported in Table 1.

**Table 1:** Sociodemographic and clinical characteristics of the sample.

Sample characteristics	Gender		Age (years)	TMJ pain	TMJ noise	Clenching
	Male [*N* (rate%)]	Female [*N* (rate%)]	Mean age (years) ± SD	Rate % [*N* (rate%)]	Rate % [*N* (rate%)]	Rate % [*N* (rate%)]
Total (*N* = 105)	46 (43.8)	59 (56.2)				
Tinnitus (*N* = 53)	23 (43.4)	30 (56.6)	49.51 ± 12.85	9 (16.98)	47 (88.68)	51 (96.22)
w/o Tinnitus (*N* = 52)	23 (44.2)	29 (55.8)	51.48 ± 10.44	45 (86.54)	35 (67.30)	30 (57.69)

Regarding clinical characteristics of the sample (Table 1), patients in the ST group showed a significantly higher prevalence of TMJ Noise (88.68%) and Bruxism (96.22%); otherwise, only16.98% experienced TMJ pain. In contrast, in the w/o ST group, the prevalence of TMJ noise was lower (67.30%), while TMJ pain and bruxism were reported by 86.54% and 57.69% of participants, respectively.

**Figure 2:**
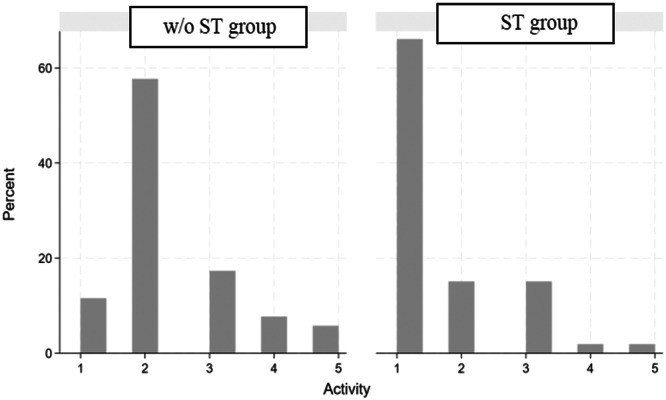
Distribution of muscle abnormal values of w/o ST group (patients with TMD but without Tinnitus) and the ST group (patients with TMD and Tinnitus). Y‐axis: percentage of subjects in each group; X‐axis: abnormal values scored from 1 to 5.

The distribution of abnormalities in the muscular activity of the masticatory muscles during the habitual resting position is represented in the two histograms shown in Figure 2. For the ST group, more than 60% of the subjects obtained a score of 1, which indicates a physiological muscle activity in the habitual rest position. By contrast, almost 60% of the subjects in the w/o ST group obtained a score of 2, which means that most of the subjects belonging to this group presented an altered activity of one of the masticatory muscles in the habitual rest position.

Regarding scores 3, 4, and 5, the percentage of abnormality was always greater in the w/o ST group than in the ST group. In fact, the average abnormality value for the ST group was 1.58 ± 0.95 and 2.38 ± 0.99 for the w/o ST group, as shown in Table 2.

**Table 2:** Levene's test results.

Levene's test	Mean ± SD	Frequency	*p*‐value
W/o Tinnitus Group	2.38 ± 0.99	52	
Tinnitus Group	1.58 ± 0.95	53	
Total	1.98 ± 1.05	105	0.935

*Note: p* value < 0.05 was considered as statistically significant.

Furthermore, once it had been demonstrated by Levene's test that the data collected from the two groups had equal variance (*p* = > 0.05), it was possible to perform an independent *t*‐test with equal variance **(**Table 3), which showed a statistically significant difference (*p* = 0.0001) and a Choen's d effect size of 0.82 between the two groups and a confidence interval between 1.32 and 1.85 for the ST group and between 2.11 and 2.66 for the w/o‐ST group.

**Table 3:** *t*‐test results.

Two‐sample *t*‐test with equal variances	Mean ± SD	Std. err.	95% CI	*p*‐value	Effect size (Cohen's *d*)
W/o Tinnitus Group (*n* = 52)	2.38 ± 0.99	0.14	[2.11−2.66]		
Tinnitus Group (*n* = 53)	1.58 ± 0.95	0.13	[1.32−1.85]		
Combined (*n* = 105)	1.98 ± 1.05	0.10	[1.78−2.18]		
Δ (w/o Tinnitus – Tinnitus)	0.80 ± 1.37	0.19	[0.42−1.18]	**0.0001** *	**0.82**

* *p*‐value < 0.05 was considered as statistically significant and are in bold.

The null hypothesis is therefore rejected, and the alternative hypothesis indicating a different behavior of the muscular activity of the patients with ST compared to the group without ST is accepted. Specifically, patients within the ST group presented reduced abnormal muscle activity at the habitual rest position compared to patients within the w/o‐ST group.

## 4 Discussion

The main result of our study was represented by higher incidence of masticatory muscle activity at rest in patients with TMD alone, compared to those with associated ST. The difference between the TMD groups with and without tinnitus was statistically significant (*p* = 0.0001), clinically relevant. The effect size (Cohen's d = 0.82) indicates a large magnitude of difference, suggesting that the presence of tinnitus is meaningfully associated with increased muscular abnormality in patients with TMD (Sullivan and Feinn 2012).

TMDs cause abnormal electrical activity of the masticatory muscles, due to the disorder itself or compensatory mechanisms secondary to symptoms (Santana‐Mora et al. 2009). When combined with the patient's clinical history and physical examination, EMG can provide objective and reproducible data on the function of masticatory muscles in patients with TMD (Hugger et al. 2012). Many studies have investigated muscle activity in patients with TMD, revealing less muscle symmetry (Mapelli et al. 2016). Higher muscle electrical activity at rest and lower in function, as well as more fatigue and unilateral mastication, have been reported in patients with TMD (Cardoso et al. 2020). Studies evaluating the masticatory tendency in patients with TMD showed a greater impairment of the masticatory function, as well as the preference side of mastication, suggesting that the masticatory tendency was a physiological defence mechanism to avoid more aches (Santana‐Mora et al. 2009; De Felício et al. 2013; Ferreira et al. 2014).

Tinnitus is a multifaceted condition with various etiologies, including auditory and nonauditory factors. While peripheral causes such as cochlear damage from noise exposure or ototoxic medications are well‐documented (Ganz Sanchez et al. 2002; Zieliński et al. 2019), emerging evidence highlights the significance of somatosensory influences, particularly those involving the TMJ and masticatory muscles (De La Torre Canales et al. 2024; Prado et al. 2024; Bernhardt et al. 2011). Nevertheless, there are only a few electromyography studies of masticatory muscles in patients with tinnitus. Increased masticatory muscle activity at rest, as observed in TMD patients, may lead to sustained muscle tension and altered proprioceptive inputs, potentially affecting auditory perception. Increased masticatory muscle activity at rest, as observed in TMD patients, may lead to sustained muscle tension and altered proprioceptive inputs, potentially affecting auditory perception (Szyszka‐Sommerfeld et al. 2023). In the study of Zieliński et al. (2019) the muscular activity in the temporomandibular muscles was examined by way of sEMG at rest, when the teeth were clenched and when the mouth was opened maximally, to assess muscular asymmetry. The authors concluded that there was a relationship between changes in the asymmetry index of the masticatory muscles and the occurrence of tinnitus, suggesting that a change in activity that does not cause pain in the muscle of mastication could also be associated with tinnitus. Nonetheless, they recommended further examination in this area and longer observation time to confirm the results (Zieliński et al. 2019).

## 5 Limitations of the Study

Despite the methodological rigor applied in this study, several limitations should be acknowledged. The sample size may not be sufficient for generalizing the findings, although the a priori sample size calculation indicated that at least 40 subjects per group would be sufficient, the relatively limited number of participants (53 in the ST group and 52 in the w/o‐ST group) may still not be adequate to generalize the findings to the entire population with TMD and ST. Larger studies are needed to improve reliability. The lack of follow‐up avoids determining how the relationship between TMD and ST evolves or whether specific therapeutic interventions could influence clinical progression. A longitudinal study could provide deeper insights into the progression of TMD and ST. The absence of a healthy control group limits the interpretation of results, as comparisons were only made between TMD patients with and without ST. Including a control group could offer clearer reference data. Lastly, the study did not assess tinnitus severity (THI [Tinnitus Handicap Inventory] and TFI [Tinnitus Functional Index]), limiting insights into its impact on quality of life. Considering these limitations, future research should aim to include larger sample sizes, longitudinal follow‐ups, healthy control groups, and more refined methodologies to deepen our understanding of the relationship between TMD and ST.

## 6 Conclusions

Neuroplasticity appears to play a significant role in modulating the psychoacoustic characteristics of tinnitus, suggesting that the development of ST may result from abnormal interactions between sensory modalities, sensorimotor systems, and neurocognitive and neuroemotional networks (Ralli et al. 2017). The hypothesis that neuromuscular dysfunction of the masticatory muscles may trigger alterations in the sound conducting apparatus (Myrhaug 1964) was not confirmed by another study reporting that spasm in the masticatory muscles of TMD patients neither seemed to cause reflex spasm of the tensor palatini muscles nor to have effect significant alterations in Eustachian tube function (Penkner et al. 2000).

A better knowledge of possible connections between changes of mastication activity and tinnitus may help in the acceleration of clinical diagnostics and tinnitus treatment (Zieliński et al. 2019).

## Author Contributions

Conceptualization: Alberto Maria Cappellari and Henri Albert Didier. Methodology: Alberto Maria Cappellari, Henri Albert Didier, and Federica Di Berardino. Software: Alexandre Henri Didier and Gennaro Bussone. Data curation: Margherita Donelli and Xi Xi Fan. Writing – original draft preparation: Alberto Maria Cappellari. Writing – review and editing: Alberto Maria Cappellari, Henri Albert Didier, Federica Di Berardino, and Margherita Donelli. Visualization: Aldo Bruno Giannì, Alberto Caprioglio, Giorgio Lilli, Stefania Barozzi, Laura Magnoni, Diego Zanetti, and Flavio Arnone. Supervision: Alberto Maria Cappellari, Henri Albert Didier, and Federica Di Berardino. All authors have read and agreed to the published version of the manuscript.

## Conflicts of Interest

The authors declare no conflicts of interest.

## Data Availability

The data presented in the study are available on request.
